# Methodologies for Analyzing Soluble Organic Compounds in Extraterrestrial Samples: Amino Acids, Amines, Monocarboxylic Acids, Aldehydes, and Ketones

**DOI:** 10.3390/life9020047

**Published:** 2019-06-06

**Authors:** Danielle N. Simkus, José C. Aponte, Jamie E. Elsila, Eric T. Parker, Daniel P. Glavin, Jason P. Dworkin

**Affiliations:** 1 NASA Postdoctoral Program at NASA Goddard Space Flight Center, Greenbelt, MD 20771, USA; 2 Solar System Exploration Division, NASA Goddard Space Flight Center, Greenbelt, MD 20771, USA; jose.c.aponte@nasa.gov (J.C.A.); jamie.e.cook@nasa.gov (J.E.E.); eric.t.parker@nasa.gov (E.T.P.); daniel.p.glavin@nasa.gov (D.P.G.); jason.p.dworkin@nasa.gov (J.P.D.); 3 Department of Chemistry, Catholic University of America, Washington, D.C. 20064, USA

**Keywords:** soluble organics, extraterrestrial samples, carbonaceous chondrites, amino acids, analytical chemistry, astrochemistry, astrobiology

## Abstract

Soluble organic compositions of extraterrestrial samples offer valuable insights into the prebiotic organic chemistry of the solar system. This review provides a summary of the techniques commonly used for analyzing amino acids, amines, monocarboxylic acids, aldehydes, and ketones in extraterrestrial samples. Here, we discuss possible effects of various experimental factors (e.g., extraction protocols, derivatization methods, and chromatographic techniques) in order to highlight potential influences on the results obtained from different methodologies. This detailed summary and assessment of current techniques is intended to serve as a basic guide for selecting methodologies for soluble organic analyses and to emphasize some key considerations for future method development.

## 1. Extraterrestrial Organic Matter

For most extraterrestrial samples, the organic content can be divided into two main components: (1) insoluble organic matter (IOM)―a macromolecular fraction that is insoluble in organic solvents [1]; and (2) soluble organic matter (SOM)―a solvent-soluble fraction that may contain a diverse range of prebiotic organic compounds, including amino acids, amines, carboxylic acids, aldehydes, ketones, alcohols, sugars, nucleobases, and aliphatic and aromatic hydrocarbons [2,3,4]. These biologically relevant molecules are synthesized by abiotic reactions during the early evolution of the pre-solar cloud and protoplanetary disk, and during subsequent photochemical-, energetic particle-, thermal and aqueous alteration-driven reactions in the solar system [5]. Extracting and analyzing soluble organic compounds from extraterrestrial samples and investigating the chemical processes involved in their synthesis brings us closer to understanding the origin of prebiotic organic molecules in the early solar system, and how these species may have contributed to the origin of life on Earth and possibly elsewhere.

## 2. Sample Types

### 2.1. Meteorites

The majority of soluble organic analyses of extraterrestrial materials are carried out on meteorites―rocky fragments of extraterrestrial bodies that have landed on the surface of a planetary body. Most meteorites originate from asteroids or comets, while a smaller number are derived from fragments of planets or moons that were ejected from their parent surfaces during large impact events. Based on their bulk compositions and textures, meteorites can be subdivided into two main categories: non-chondrites and chondrites [6]. The non-chondrites, which include the achondrites, stony–iron meteorites, and iron meteorites, have experienced partial or complete melting and planetary differentiation. In contrast, chondrites are derived from primitive, undifferentiated asteroids or comets, and their contents may represent some of the oldest materials in our solar system. They are generally characterized by the presence of chondrules―small, round olivine- and pyroxene-rich grains that formed as molten or partially-molten droplets during early solar system formation. Due to their primitive nature and often comparatively high (~3%) abundances of organic matter, chondrites are the most commonly studied meteorite samples for soluble organic analyses. Of the five mineralogic classes of chondrite meteorites (carbonaceous, ordinary, enstatite, R (Rumuruti-like), and K (Kakangari-like)), some of the most carbon- and soluble organic-rich meteorite groups belong to the carbonaceous chondrites (CI, CM, CR, CO, CB, CH, CV, CK and C_ungrouped_ chondrites) and they constitute the bulk of the meteorite materials analyzed for soluble organics. The letters designating the categories of carbonaceous chondrites represent typical chondrites within each group (CI―Ivuna; CM―Mighei; CR―Renazzo; CO―Ornans; CB―Bencubbin; CV―Vigarano; CK―Karoonda), with the exception of the CH group which is assigned the letter ‘H’ in reference to its high metal and iron content. The C_ungrouped_ category represents meteorite specimens with unique mineralogical and chemical properties that currently do not have enough known specimens to warrant the creation of a new class. Following the mineralogic class, a petrologic type is often assigned to indicate the relative degree of parent body alteration, with types 1–3 having experienced aqueous alteration at low temperature (1 = most aqueously altered; 3 = least aqueously altered) and types 4– 6 having experienced varying levels of thermal alteration (4 = least thermally altered; 6 = most thermally altered) [7].

The first published report of organic matter within a carbonaceous chondrite was a study of the Alais CI1 carbonaceous chondrite in 1834 [8]. The earliest investigations of the chemical composition of soluble organic matter in carbonaceous chondrites began with bulk carbon measurements of solvent extracts, and analyses of hydrocarbons and amino acids, with some uncertainties distinguishing between terrestrial and extraterrestrial origins (e.g., [9,10,11,12]). The fall of the Murchison CM2 carbonaceous chondrite in 1969 and, subsequently, the first measurements of non-protein amino acids in Murchison [13] led to greater scientific interest in meteoritic organics and significant improvements in analytical sensitivity. The database of known soluble organic compositions quickly expanded from only a handful of compounds to large datasets comprised of isomers and stereoisomers of multiple classes of soluble organics (e.g., [14,15,16,17,18]).

#### Obtaining Meteorite Samples for Analysis

**Figure 1:**
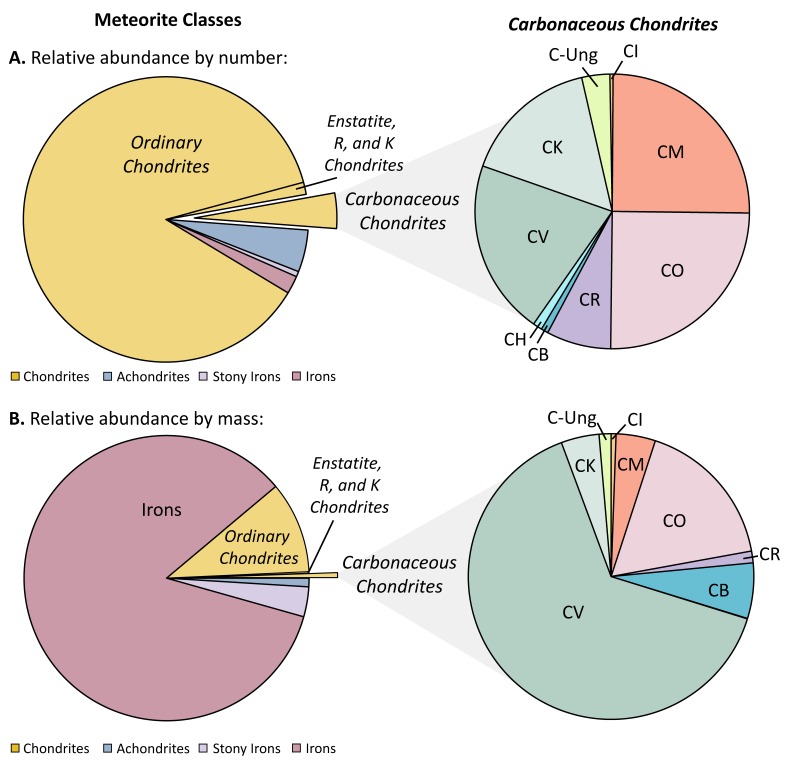
Relative abundances of chondrites, achondrites, stony iron meteorites and iron meteorites, and of individual chondrite groups, based on (**A**) total numbers of meteorites (falls + finds) identified for each category and (**B**) total mass of meteorite material available from each category. Relative proportions are approximate values based on data obtained from the Meteoritical Bulletin Database [21] (March 2019).

Meteorite falls occur all across the Earth’s surface. However, the vast majority of meteorites are collected in hot and cold desert environments (e.g., the Sahara and Antarctica, respectively) where dark meteorite fragments are easily distinguishable across vast regions of light-colored terrain with minimal surface features that would otherwise inhibit detection of these extraterrestrial specimens. Furthermore, cold-arid environments, such as Antarctica, provide an added benefit of exposing meteorites to minimal terrestrial alteration and contamination. Certain regions along the Transantarctic Mountains often harbor large numbers of meteorites because meteorites are naturally transported across the continent within vast sheets of flowing glacier ice and become concentrated along mountain ridges, which can act as natural barriers. Meteorites are annually collected by government-funded organizations (e.g., the Antarctic Search for Meteorites [19] (ANSMET) and the Japanese Antarctic Research Expedition [20] (JARE) programs), as well as by professional meteorite collectors and amateurs. Although there are currently > 60,000 meteorites available for study [21], the most organic-rich and scientifically valuable specimens for soluble organic analyses are relatively few in number. Chondrites comprise the largest proportion of meteorites known to date (~92%, based on number of meteorites) but the bulk of the group is comprised of relatively organic-poor samples (ordinary chondrites, which constitute ~87% of all meteorites, by number (Figure 1; [21]). Carbonaceous chondrites constitute only ~4% of all meteorites identified and represent ~0.5% of the total mass of meteorite material available. In terms of alteration history, the end-member groups also have the fewest number of samples. For instance, CI carbonaceous chondrites―some of the most highly aqueously altered and most primitive in terms of bulk elemental compositions―are represented by only nine known examples to date. Six of the CIs have a total available mass of only a few grams, and two of the CIs (Alais and Revelstoke) are not available for destructive analysis at all. For some carbonaceous chondrite groups (e.g., CIs and CVs), only one petrologic type has been observed to date. The rarity and generally low sample masses of meteorite samples that have experienced unique parent body histories makes these samples particularly desirable for soluble organic studies. For these reasons, obtaining a meteorite sample from an academic or government-owned collection often requires a formal sample request with valid justification for analyzing a specific sample mass of a particular meteorite specimen.

### 2.2. Dust and Micrometeorites

Like meteorite samples, micrometeorites (MMs) and interplanetary dust particles (IDPs) can originate from asteroids, comets, or other planetary bodies; however, they are much smaller in size (MMs ~50–500 µm; IDPs ~10–40 µm), which presents a unique analytical challenge. Most organic analyses of these samples are focused on spatially resolved techniques without the ability to differentiate between soluble compounds beyond measurements of mass (e.g., microprobe two-step laser mass spectrometry (µL^2^MS) [22,23]), bulk isotopes (e.g., nanoscale secondary ion mass spectrometry (nanoSIMS) [24]), and functional groups (e.g., x-ray absorption near edge structure (XANES) spectroscopy and infrared spectroscopy [25]). In addition to these targeted analyses of individual MMs or IDPs, bulk solvent extractions of collections of MM grains have been performed for compound-specific measurements of amino acids using methodologies commonly applied to carbonaceous chondrite samples [24,26,27,28]. For amino acid analysis, disaggregation of MMs prior to the hot-water extraction step has been carried out by crushing and sonicating MM particles in ultrapure water [24,26]. Hot-water extractions have also been carried out on intact MMs directly, without weighing or crushing the particles, in order to mitigate potential contamination of the samples [27].

### 2.3. Spaceflight Mission Samples

Unlike most meteorites, extraterrestrial samples collected by robotic or crewed spacecraft are from a known location, they offer the opportunity to collect material from a desired sample source, and potentially provide a means by which to mitigate terrestrial contamination. However, these types of samples are generally more limited in abundance and more difficult to obtain than meteorites. Typically, a researcher must demonstrate proficiency in meteorite analyses and relevant analogs of the appropriate size and class in order to credibly propose to analyze samples collected through these missions. Such samples are carefully controlled by the government that curates them (e.g., https://curator.jsc.nasa.gov/lunar/index.cfm). Soluble organic analyses of sample return material often involve applying the same techniques as those used for analyzing carbonaceous chondrites. In fact, many of these chromatographic techniques were developed or refined to analyze the lunar samples returned by the NASA Apollo 11, 12, 14–17 missions [29,30,31,32,33,34,35]. Future analyses of samples returned from asteroid Ryugu by JAXA’s Hayabusa2 mission and from asteroid Bennu by NASA’s OSIRIS-REx mission [36] are expected to apply an array of methods. Small particles from the Itokawa asteroid obtained through the Hayabusa sample return mission have been analyzed similarly to IDPs and MMs, though their compositions are relatively depleted in organics [37,38]. Alternative collection techniques (e.g., aerogels and aluminum foils exposed to the Stardust comet Wild 2 coma [39,40,41]) have been used to examine bulk material, instead of individual particles, to make compound-specific measurements via chromatographic methods such as those described here. In contrast to sample return analyses, in situ soluble organic analyses of relatively distant planetary bodies require easily transportable instrumentation with minimal mass, power, and volume requirements. Technologies in development for these purposes include, for example, capillary electrophoresis and a new miniaturized variant of the method, microchip electrophoresis, for chiral analysis of amino acids [42].

## 3. Review Objectives

**Scheme 1:**
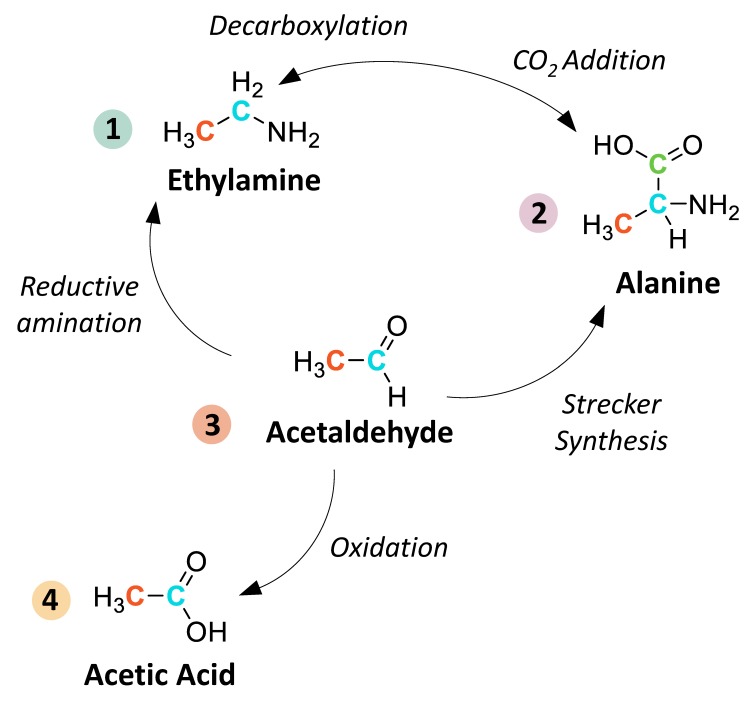
A simplified schematic of potential synthetic relationships between extraterrestrial water-soluble (“free”) amines^(1)^, amino acids^(2)^, aldehydes/ketones^(3)^, and monocarboxylic acids^(4)^. These reactions include amine synthesis via reductive amination of aldehydes/ketones [43] and decarboxylation/deamination of amino acids, carboxylic acid synthesis via oxidation of aldehydes [44], and amino acid synthesis via Strecker reactions and CO_2_ addition to amines [45]. Other potential mechanisms for amino acid synthesis not illustrated here include, for example, reductive amination of keto acids [46], β-amino acid synthesis via Michael addition of ammonia to α,β-unsaturated nitriles [17], and γ- and δ-amino acid synthesis via decarboxylation of α-amino dicarboxylic acids [47]. Bound amino acids that are released from samples via acid hydrolysis may be derived from larger organic polymers or from transition metal complexes. Detailed descriptions of these reaction mechanisms have been described elsewhere (see [5] and references therein).

In the present literature review, we aim to provide a comprehensive guide for the analysis of soluble organic compounds in extraterrestrial materials, summarizing key modifications and new techniques introduced over the past >50 years of research. Methodologies developed for terrestrial sample analyses (e.g., for pharmaceutical or environmental purposes) are not always applicable to extraterrestrial studies. The organic compositions of astromaterials are often unique to extraterrestrial environments and, due to limited sample quantities and low abundances of organics, techniques with relatively high sensitivity are required. This review focuses specifically on methodologies targeting five classes of meteoritic soluble organics that are readily extractable in water or polar solvents and that are structurally related by synthetic pathways potentially occurring inside the asteroid parent body: amino acids, amines, monocarboxylic acids, aldehydes, and ketones (Scheme 1).

A second objective of this review is to highlight observed variability between meteorite studies and discuss the potential impact of meteorite sample heterogeneity and the effects of differing analytical techniques (e.g., solvent extraction methods, derivatization methods, and chromatographic separation techniques). The ultimate goal of this discussion is to raise awareness of potential limitations to be considered during method development and new targeted analyses, in order to help mitigate ambiguity among collaborative cross-comparisons. With these considerations in mind, we provide a summary and assessment of the current state of knowledge regarding meteoritic soluble organics and discuss potential avenues for improvements during future analyses, including exploring new analytical techniques and targeting new classes of organic compounds.

## 4. Analysis of Extraterrestrial Organic Compounds: Method Overview

### 4.1. Mitigation and Monitoring of Sample Contamination

Special care must be taken prior to and during organic analyses of any extraterrestrial sample. Meteorite specimens that have been exposed to minimal terrestrial contamination and have known curatorial histories are preferable for soluble organic analyses and, when available, terrestrial environmental samples collected from the fall site should be analyzed in parallel with meteorite samples in order to assess whether there has been any terrestrial input [48,49,50,51,52,53]. As a common rule, any glassware or tools (e.g., forceps, mortar and pestle) used for sample storage, processing, and analyses are typically wrapped in aluminum foil and heated in air at or above 500 °C for several hours (usually > 6 h) prior to use. This combustion process drives off organics that could be introduced into the samples. Other measures that are commonly taken to mitigate and monitor terrestrial contamination and cross-contamination in extraterrestrial samples include the following: (1) storing and processing samples in clean laboratory conditions (e.g., cleanroom facilities, low-temperature laboratory facilities [54], high efficiency air (HEPA) filtered laminar flow hoods, and/or gaseous nitrogen or argon gloveboxes [54,55]; (2) bead-blasting reusable ceramic mortars and pestles before baking them to remove residual sample powder; (3) removing the fusion crust outer surfaces of meteorite chips before powdering samples; (4) substituting plastic tools with polytetrafluoroethylene (PTFE) materials (e.g., pipette bulbs, storage containers), and using PTFE-lined lids and caps for glass containers; (5) wearing powder-free nitrile gloves during sample handling and analyses (not latex or nylon, as these materials may degrade to form amino acids); (6) using only high purity (>99%) solvents and ultrapure water (triple-distilled, or Milli-Q^®^ (18.2 MΩ cm, <5 ppb total organic carbon)) for carrying out sample extractions and preparing solutions; (7) analyzing the cleanliness of chemical reagents prior to using them for sample processing and analysis, to measure background levels of organics present in derivatization and purification reagents, as well as in the analytical instrumentation; and (8) analyzing blanks in parallel with samples, including solvent blanks (i.e., solvent only, no meteorite sample powder) and organic-free mineral analog blanks (usually baked serpentine mineral or glass powder, sand, or meteorite powder, extracted and carried through the method). Some of these techniques (e.g., cleanroom protocols) are designed to reduce particulate contamination, without special attention to soluble organic compounds. The challenges associated with soluble organic analyses are different from those typically experienced in a cleanroom environment [56]; for example, the presence of inorganic dust is of minimal consequence when executing soluble organic analyses, but the presence of organic gases (e.g., HCN, HCHO) that are not removed by filtration steps can often pose a problem. Conversely, aluminum-wrapped glassware cleaned by heating in a ceramic muffle furnace, which is ideal for soluble organic analysis, can introduce contaminants for bulk inorganic analyses. Further details regarding specialized curation and contamination control protocols can be found in the literature (e.g., [54,56]).

Certain molecular structures and isotopic signatures may be used to assess the potential influence of terrestrial contamination. Monitoring the presence of these terrestrial signatures in addition to performing the types of procedural blank analyses described above is valuable for identifying contaminants introduced prior to sample analyses. Biological and terrestrial signatures include, for example, the presence of large l-enantiomeric excesses (“non-racemic” mixtures) of proteinogenic amino acids (e.g., serine, threonine, isoleucine, and glutamine), d-enantiomeric excesses of isovaline potentially derived from fungal peptides [57], and the presence of polymers and plasticizers (e.g., nylons, polyethylene glycol, and phthalates) and silicon-based grease. In contrast, the presence of isotopically heavy organic compounds (δ^13^C > 0‰ and δD >> 0‰, which are characteristic of extraterrestrial low-temperature gas-phase reactions [58,59,60]) can be indicative of extraterrestrial origins.

### 4.2. Typical Sample Extraction Protocols 

**Figure 2:**
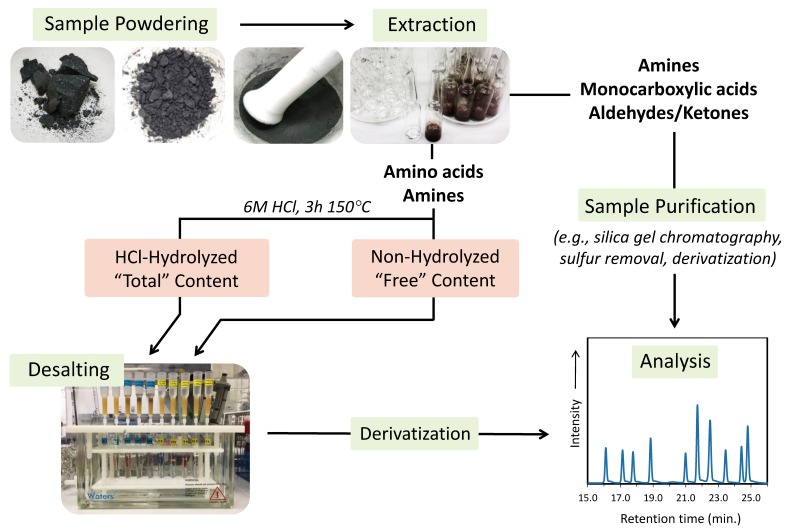
Simplified schematic of a typical protocol for extracting and analyzing amino acids, amines, monocarboxylic acids, aldehydes, and ketones in extraterrestrial materials.

The general procedure for extracting and analyzing soluble organic compounds from extraterrestrial samples is illustrated in Figure 2. Typically, a solid sample is powdered using a ceramic or corundum mortar and pestle. An agate mortar and pestle can also be used; however, as agate cannot be heated to high temperatures for cleaning, extra care must be taken to sufficiently remove organic contaminants using ultrapure water and solvents. The fine powder is then weighed and transferred to a receptacle (e.g., glass tube, ampule, or round-bottom flask) for the solvent extraction step. Polar organic compounds such as amino acids, amines, monocarboxylic acids, aldehydes, and ketones, are typically extracted in ultrapure water for varying periods of up to 24 h, commonly at 100 or 110 °C (Table 1, Table 2, Table 3 and Table 4). These hot-water extractions can be carried out using a reflux set-up or as small aliquots (1–2 mL water for 0.5–1 g of powder). A number of studies have carried out meteorite extractions of polar organic compounds in organic solvents, such as KOH/CH_3_OH mixtures or 1 N NaOH for the extraction of carboxylic acids [61,62,63,64,65], and dichloromethane (DCM) for the extraction of aldehydes and ketones [66]. Relatively non-polar organic species (e.g., hydrocarbons and heterocycles) are typically extracted using either CH_3_OH, DCM, DCM/CH_3_OH mixtures, or benzene/CH_3_OH mixtures [13,67,68,69,70,71,72,73,74]. In this review, we focus on those compounds that are readily accessible through aqueous extractions.

### 4.3. Processing and Purifying the Extract

#### 4.3.1. Separating and Concentrating the Supernatant

Following the extraction step, the solution containing the extracted organics is commonly centrifuged and/or filtered to separate the supernatant from the solid sample residue, as solids suspended in solution can interfere with subsequent derivatization or purification reactions. Rinsing the residual powder with additional solvent and repeating the centrifugation/filtering step helps to maximize the recovery of extracted compounds. Methods for concentrating the supernatant include using centrifugal vacuum concentrators, rotary evaporators, and cryodessicators, or drying with nitrogen gas. For some analyses, volatile compounds (e.g., monocarboxylic acids, amines) that may otherwise be lost from the extract during solvent evaporation are retained in the sample by adjusting the pH to convert the analytes to organic salts [65,70,75,76,77,78,79,80].

#### 4.3.2. Acid Hydrolysis

**Figure 3:**
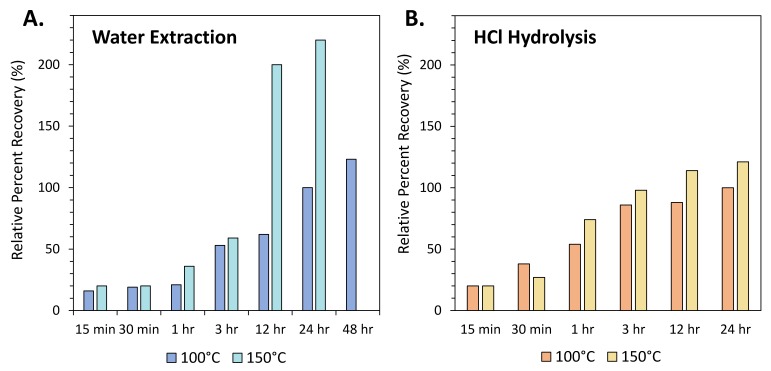
Average percent recoveries (%) of amino acids extracted from Nile Delta deep-sea sediment (see Glavin et al., 1999 [49] for sample source information), relative to the standard amino acid extraction protocol (100 °C water extraction for 24 h, followed by 6 M HCl hydrolysis at 100 °C for 24 h). (**A**) Effects of varying temperature (100 °C and 150 °C) and duration (15 min–48 h) for the hot-water extraction. Increasing the duration and the temperature of the water extraction results in a continuous increase in amino acid recovery. (**B**) Effects of varying temperature (100 °C and 150 °C) and duration (15 min–24 h) for 6 M HCl hydrolysis. In most cases, increased temperature and duration results in higher amino acid recoveries; however, the duration of the hydrolysis step is generally kept at 3 h to avoid potential amino acid racemization [89].

A meteorite extract may be carried through an acid hydrolysis step to hydrolyze and release organic molecules that may be bound as larger polymers or complexed with transition metal species within the meteorite [81]. This process may also produce amino acids from precursor molecules, including amino esters, amides, amino nitriles and lactams [47]. Acid hydrolysis is most commonly carried out for amino acid analysis (Table 1, Figure 2), but has also been used for the analysis of meteoritic amines (e.g., [15,82]). The acid-hydrolyzed fraction of a meteorite extract often exhibits a distinct composition and, in many cases, comprises the bulk of the amino acid content (e.g., [50,83,84]). The earliest hydrolysis experiments involved adding acid directly to the dried water extract residue [85,86,87]. This procedure was later modified to minimize the potential for contamination during direct liquid hydrolysis by optimizing a vapor-hydrolysis technique [88]. The duration of the hydrolysis step was also reduced to 3 hours in order to minimize the potential for amino acid racemization (Figure 3; [49]). Carrying out acid hydrolysis at higher temperatures and for longer durations has been shown to lead to significant racemization of chiral amino acids [89].

#### 4.3.3. Purification Methods

Sample purification steps are commonly carried out to remove impurities and isolate soluble organic compounds of interest for chromatographic analysis. Common purification techniques include treating the meteorite extract with activated silica gels (e.g., [65,82]), performing liquid-liquid extractions to isolate specific target molecules (e.g., [61,63]) and removing co-extracted elemental sulfur from solution using copper powder or granules (e.g., [66,70]). These purification steps may be carried out before or after derivatization, depending on the extraction procedure, target analytes, and derivatization chemistry. For amino acid analysis, passing the aqueous extract through an ion exchange chromatography column (“desalting”, Figure 2) prior to derivatization helps remove inorganic salts that may otherwise interfere with subsequent derivatization reactions and/or damage gas chromatography (GC) or liquid chromatography (LC) columns. Given the importance of desalting for the analyses of meteoritic amino acids, a detailed description of this purification effort, what types of ion exchange approaches are typically used, and how to minimize contamination when executing the desalting step, are discussed here.

Ion exchange chromatography provides separation of molecules based on charge by utilizing resins that are typically composed of beads of polymers, with electrolytes covalently bound to the resin beads ([90], and references therein). One of the ions of the electrolyte is fixed (“stationary”) to the resin beads, while the counterion is of the opposite charge and is capable of being exchanged (i.e., is “mobile”). During sample elution through the chromatography column, compounds that are neutral, or possess an identical charge to the stationary ion, are eluted through the column. In contrast, sample molecules with a charge opposite to the stationary ion, compete with the mobile ions for binding sites on the resin beads. Sample ions that have a greater affinity for the stationary ions are capable of displacing mobile ions to become bound to the resin beads. Bound sample ions can then be selectively eluted from the chromatography column using an eluant with an appropriate pH and ionic strength, such that the eluent contains either (A) an ion with a greater affinity for the stationary ions than do the sample ions, or (B) an ion with a selectivity for the stationary ions that is equal to, or less than, that of the sample ions, but is present at a high concentration.

For analyses of amino acids in meteorites, cation exchange chromatography has proven beneficial for removing inorganic salts and other interfering species, while concomitantly enabling the elution of amino acids in a solution that does not complicate their subsequent analyses [91]. Cation exchange chromatography resins that are typically used when preparing meteorites for amino acid analyses include AG 50W-X8, 100–200 mesh, hydrogen form (Bio-Rad Laboratories, Hercules, CA, USA [50]), and AG 50W-X4, 200–400 mesh, hydrogen form (Bio-Rad Laboratories, Hercules, CA, USA [92]). These resins have a SO_3_^−^ stationary ion fixed to the beads, and an H^+^ mobile ion bound to the stationary ion. When a meteorite extract containing amino acids is loaded onto the chromatography column, the R-NH_3_^+^ ions of the amino acids will outcompete the H^+^ mobile ions for binding sites, and thus displace the mobile ions to become bound to the resin beads. Afterward, the amino acids can be selectively eluted from the resin typically using 2 M NH_4_OH.

Prior to use, cation exchange chromatography columns are typically rinsed to minimize potential sources of contamination ([93], and references therein). First, the columns are rinsed with 6 bed volumes of ultrapure water (until the eluting solution has a neutral pH). Second, a base wash is executed to remove the H^+^ mobile ions originally present on the resin using 4 bed volumes of 2 M NaOH (until the eluting solution has a basic pH). Third, the chromatography columns are rinsed with 12 bed volumes of ultrapure water (until the eluting solution has a neutral pH). Fourth, the resin is recharged with fresh H^+^ mobile ions using 2 bed volumes of 1.5 M double distilled hydrochloric acid (ddHCl) (until the eluting solution has an acidic pH). Finally, the chromatography columns undergo a final water rinse using 12 bed volumes of water (until the eluting solution has a neutral pH).

There are certain important considerations to bear in mind when preparing and using ion exchange chromatography columns, including: (1) Do not allow resins to dry, which may allow cracks to form, and (2) The water rinses performed after the NaOH wash and the ddHCl recharging steps are important to execute in order to return the columns to a neutral pH environment without exposing the columns to a pH shock that may otherwise crack the resins.

### 4.4. Chromatographic Separation and Detection

The separation and detection of meteoritic soluble organics is most commonly performed using gas chromatography-mass spectrometry (GC-MS), or high-performance liquid chromatography coupled to a UV fluorescence detector (HPLC-FD), and/or a mass spectrometer (e.g., time-of-flight mass spectrometer (ToF-MS)). Both GC and LC separation techniques offer different analytical advantages, and certain meteoritic organic compounds have been detected using only one method and not the other. GC is generally used for analyzing relatively volatile, low molecular weight, and thermally stable compounds, while LC techniques are more appropriate for analyzing non-volatile and thermally unstable analytes. The chromatographic separation technique best suited for a particular study is also dependent on the concentrations of the analytes in the sample and the level of instrumental sensitivity needed. Typically, LC methods using a fluorescent detector are one to a few orders of magnitude more sensitive than GC methods. On the other hand, if stable isotopic measurements are needed and sufficient sample mass is available, then GC protocols are followed. Nevertheless, LC and GC methods can be mutually beneficial; a small fraction of a sample can be analyzed using LC as an initial assessment of concentrations to provide feedback for further LC or GC analyses.

Detection methods vary depending on the chromatographic separation technique used. For GC analysis, parent molecules are typically ionized via electron impact ionization, and characteristic fragmentation patterns can be compared to mass spectral libraries for compound identification. LC techniques are more amenable to parallel detection methods (e.g., optical/fluorescence detection and mass spectrometry). Positive electrospray ionization is the most common ionization method for LC due to high sensitivity. Unlike electron impact ionization, the electrospray method results in minimal mass fragmentation. Other fragmentation methods within the ion optics of the instrument are possible to gain structural information, and different mass analyzers (e.g., quadrupole, time-of-flight, ion trap, etc.) provide different resolutions and scan speeds. A more thorough review of mass spectrometry is outside the scope of this manuscript.

While some compound classes can be separated chromatographically as underivatized samples (e.g., GC analysis of monocarboxylic acids via direct solvent injection or solid-phase micro-extraction (SPME)), most meteoritic analyses involve an intermediate chemical derivatization step to convert the target analytes to more GC- or LC-amenable derivatives. Table 1, Table 2, Table 3 and Table 4 summarize the range of chromatographic methods that have historically been used for analyses of meteoritic amino acids, amines, monocarboxylic acids, aldehydes, and ketones. In the following sections, we discuss the various methodologies used, with an emphasis on the relatively recent techniques. While amino acid analyses have been successfully carried out using a wide range of methods, the number of techniques used for analyses of other compound classes is relatively limited.

#### 4.4.1. Amino Acids

**Scheme 2:**
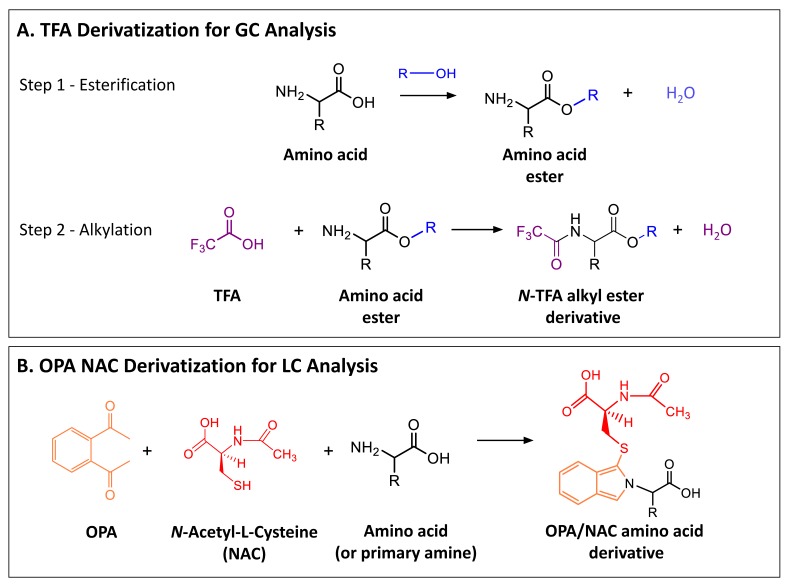
Schematic of the (**A**) trifluoroacetic acid (TFA) derivatization reaction and (**B**) *o*-phthalaldehyde-*N*-acetyl-l-cysteine (OPA/NAC) derivatization reaction commonly used for analyses of meteoritic amino acids.

The large majority of reports of aliphatic amino acids in extraterrestrial samples can be grouped into two main categories based on methodologies for derivatization and analysis: (1) analyses using trifluoroacetic acid (TFA) or trifluoroacetic anhydride (TFAA) derivatization for GC analysis (Scheme 2, panel A); and (2) analyses using *o*-phthalaldehyde-*N*-acetyl-l-cysteine (OPA/NAC) derivatization for LC (Scheme 2, panel B). The TFA/TFAA derivatization method involves esterification by reaction with an alcohol, followed by alkylation of the amino acid ester (Scheme 2, panel A). Two enantiomers of a chiral amino acid can be separated if a chiral alcohol is used for the esterification step (e.g., (*S*)-(+)-2-pentanol [86]) and/or if a chiral GC-column is used for the separation of derivatives. For OPA/NAC, the reaction between chiral amino acids and the chiral thiol N-acetyl-l-cysteine produces two diastereomers (representing the two amino acid enantiomers) (Scheme 2, panel B) which can be separated using non-chiral reversed phase LC columns. Other methodologies used for chiral separation of meteoritic amino acids include the use of enantioselective chiral columns for GC analysis of *N*-ethoxycarbonyl ethyl ester (ECEE) and *N*-ethoxycarbonyl heptafluorobutyl ester (ECHFBE) derivatives [94,95], GC analysis of amino acids as *n*-pentafluoropropionic anhydride (PFP) alkyl ester diastereomers [96,97,98], and the use of a chiral zwitterionic surfactant for micellar electrokinetic chromatography of Pacific Blue succinimidyl ester (PBSE) derivatives [99]. Further details regarding both enantiomeric and stable isotopic analyses of meteoritic amino acids are reviewed in the literature (e.g., [91,100,101,102,103,104]).

The TFA/TFAA derivatization method for GC analysis has proven to be particularly advantageous in allowing a wide range of C_6_ and C_7_ amino acid TFA derivatives to be separated and detected [105,106]. For OPA/NAC derivatization, the introduction of smaller column resins and higher pressures (ultra high precision liquid chromatography (UHPLC)) and the use of UV fluorescence detection coupled with ToF or other high resolution mass spectrometry [50] was uniquely advantageous in allowing individual amino acids to be simultaneously identified based on their characteristic chromatographic retention times, their fluorescence absorbance patterns, and their accurate masses. This analytical approach provided greater accuracy for compound identification compared to chromatography or mass spectrometry approaches, alone. Despite these advantages, there are some known limitations associated with compound selectivity and/or chromatographic co-elutions. For instance, the basic proteinogenic amino acids (arginine, histidine, and lysine) are not detectable using either TFA/TFAA, or OPA/NAC derivatization approaches [107,108,109,110]. Therefore, these techniques are insufficient for determining if basic amino acids are present in chondrite extracts. Other derivatization methods (e.g., “Accq∙TAG” [56,111,112]) can detect these species but cannot discriminate enantiomers. Likewise, some unknown meteoritic organics are potentially masked by chromatographic co-elutions in conventional one-dimensional chromatography approaches. For example, glutamic acid and pyroglutamic acid are known to coelute with one another when implementing one-dimensional GC, but can be resolved when using novel two-dimensional GC×GC techniques [95].

#### 4.4.2. Amines

The earliest report of individual meteoritic amines [15] compared measurements from three analytical techniques: GC-MS analysis of both free underivatized amines and 2,4-dinitrophenyl (DNP) amine derivatives, and ion exchange chromatographic analysis of amine OPA derivatives. Low molecular weight aliphatic monoamines were detected; however, unambiguous identifications were not possible for several of the compounds due to incomplete chromatographic separation. As shown in Table 2, subsequent analyses of meteoritic amines were performed with improved GC techniques that incorporated *N*-pentafluoropropionic anhydride (PFPA) derivatization [113] and trifluoroacetic anhydride (TFA) derivatization techniques [76,114,115,116,117]. A derivatization method using an enantiopure reagent ((*S*)-(–)-*N*-(trifluoroacetyl) pyrrolidine-2-carbonyl chloride; TPC) was later introduced to allow for both enantiomeric and compound-specific stable isotopic analyses (δ^13^C) of meteoritic amines [82].

#### 4.4.3. Monocarboxylic Acids

Meteoritic monocarboxylic acids are most often analyzed via GC-MS as underivatized compounds, either through direct solvent injection [74,76,115,117,118] or using SPME [70,75,77,78,80,119,120] (Table 3). Avoiding sample derivatization decreases sample processing time, minimizes sample transfer steps which may otherwise result in partial loss of relatively volatile acids, and reduces the risk of introducing contamination. Using the SPME method, target analytes can be extracted from a filtered aqueous meteorite extract onto a solid coated fiber, which is then injected directly into the GC injection port. This method is particularly beneficial in eliminating the need for a liquid-liquid extraction step (e.g., water–CH_2_Cl_2;_ [61,63]) which could potentially result in a loss of volatile, water-miscible, low molecular weight acids. Despite these advantages, however, complete chromatographic separation of some chiral monocarboxylic acids is more readily achieved using an intermediate derivatization step. For chiral analysis of monocarboxylic acids, novel derivatization methods have been developed and applied (e.g., (*S*)-1-phenylethyl and (*S*)-(–)-2-methylbutanol ester derivatives [65,79]; Table 3) to minimize co-elutions that would otherwise hinder accurate chiral quantification and simultaneous compound-specific δ^13^C analysis, respectively. 

#### 4.4.4. Aldehydes and Ketones

Individual aldehydes and ketones were first identified in meteorite samples via GC-MS as underivatized compounds, and by colorimetric analysis as 3-methyl-2-benzothiazolone hydrazine hydrochloride (MBTH) derivatives [14]. The Environmental Protection Agency (EPA) method #556, which uses 2,3,4,5,6-pentafluorobenzyl hydroxylamine (PFBHA) as the derivatization reagent for aldehyde and ketone analysis, was later applied to the analysis of these carbonyl species in several carbonaceous chondrites [74,114,115,119,121] (Table 4). The PFBHA derivatization method results in the formation of two derivatives ((*E*)- and (*Z*)-isomers) for all aldehydes and ketones that have asymmetric chemical structures. For most low molecular weight aldehydes and ketones, the generation of two PFBHA-derivatives does not pose a problem for chromatographic separation and quantification. However, any resulting partial co-elutions between the isomer peaks may complicate measurements, and the conversion of a single aldehyde or ketone to produce two separate derivatives could potentially result in isotopic fractionations. As such, a novel derivatization method ((*S*,*S*)-(–)1,4-dimethoxy-2,3-butanediol (DMB) derivatization [66] has recently been developed for performing accurate δ^13^C analysis without the aforementioned potential influences of the PFBHA method (Table 4).

## 5. Identifying Limitations of Cross-Comparisons between Studies

Studies of meteoritic organics rely heavily on cross-comparisons between studies, largely due to sample availability limitations. As a whole, this process of comparing and contrasting values between reports is practical and informative. However, as the field begins to more thoroughly investigate small variations in reported values―for instance, comparing relative abundance measurements of individual compounds in chondrites to infer relative degrees of parent body alteration―it becomes increasingly important to understand how several factors (e.g., meteorite sample heterogeneity and differences in laboratory procedures) can influence the measurements. The main objectives of the following sections are to highlight potential influences on reported values, emphasizing the need for detailed method development, method comparisons, and careful reporting of meteorite specimen information. The intent of this discussion is not to discredit data comparisons across different studies but to caution that there are several potential sources of variability to consider for future research. 

### 5.1. Meteorite Sample Heterogeneity

**Figure 4:**
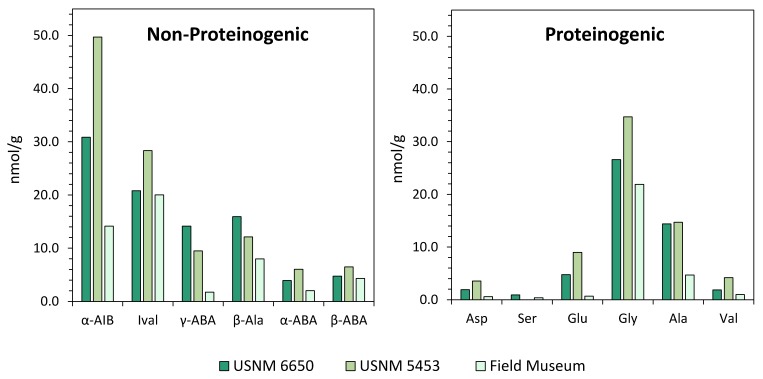
Compositional heterogeneity among Murchison meteorite specimens. Abundances (nmol/g) of non-proteinogenic and proteinogenic amino acids in three Murchison specimens (United States National Museum (USNM) 6650 [50]; USNM 5453 [122]; specimen from the Field Museum of Natural History, Chicago [125]) analyzed using the same methodology (*o*-phthalaldehyde-*N*-acetyl-l-cysteine (OPA/NAC) derivatization and high-performance liquid chromatography coupled to a UV fluorescence detector (HPLC-FD) and liquid chromatography (LC)- time-of-flight mass spectrometer (ToF-MS)) within the same laboratory facility. Abundances reported in ng/g [50,122] were converted to nmol/g values for cross-comparison. α-AIB: α-aminoisobutyric acid; Ival: isovaline; γ-ABA: γ-amino-*n*-butyric acid; β-Ala: β-alanine; α-ABA: α-amino-*n*-butyric acid; β-ABA: β-amino-*n*-butyric acid; Asp: aspartic acid; Ser: serine; Glu: glutamic acid; Gly: glycine; Ala: alanine; Val: valine.

For soluble organic analyses, the Murchison meteorite is one of the most widely studied carbonaceous chondrites in the literature, due to the large quantity of sample material available for analysis, its relatively high abundance of soluble organic compounds, and the reasonably pristine nature of the meteorite. Like most meteorites, Murchison is generally assumed to exhibit a low compositional heterogeneity; however, there have been reports of significant discrepancies in soluble organic compositions across different Murchison specimens [105,122]. These variations potentially reflect heterogeneity within the asteroid parent body, contributions from terrestrial sources, and/or loss of organics through sample weathering. Like many fallen meteorites, the Murchison fragments landed across many kilometers within a strewn field [123] and consequently, likely experienced differing levels of fracturing and weathering [124]. This potential variability between meteorite specimens should be taken into consideration for cross-comparisons between different analyses. Figure 4 illustrates a specific case study of observed compositional heterogeneity for Murchison meteorite specimens. The dataset is split into non-proteinogenic and proteinogenic amino acid distributions to illustrate intrinsic sample heterogeneity versus variations potentially induced by terrestrial inputs. Alpha-aminoisobutyric acid (α-AIB) shows the largest variation among the non-proteinogenic amino acids, as has been demonstrated previously [105]. The larger variability seen for some non-proteinogenic amino acids, such as α-AIB and isovaline, may be explained by their greater steric hindrance, which can inhibit reaction efficiencies with derivatization reagents, potentially leading to more quantitation errors.

Improved analytical sensitivity over the years has allowed sample sizes for amino acid analyses to be reduced from several grams in the 1960s to milligram and microgram quantities for present-day analyses (e.g., [126,127]). As a result, over time, meteoritic amino acid analyses have become more targeted and potentially less representative of the bulk average composition. Several recent investigations have aimed to assess potential small-scale heterogeneity of organic matter in meteorites and have identified organic compounds distributed within the fine-grained mineral matrix [127,128,129] possibly in association with altered mineral grains [130]. As such, differing sample sizes may be an important consideration for comparisons between earlier datasets and more recent analyses. In addition, the rarity and generally limited sample availability of carbonaceous chondrites restrict the number of analyses that can be carried out for each individual meteorite specimen. More often than not, a manuscript will report and discuss the soluble organic content of only one or a few meteorite specimens with comparisons to datasets reported by other studies. Across research groups, different methodologies (e.g., different extraction procedures or derivatization reactions) can produce small variations in reported values (Section 5.2), which may complicate comparisons across studies.

### 5.2. Influence of Varying Methodologies

#### 5.2.1. Influence of Extraction and Sample Processing

The types and abundances of soluble organic compounds extracted from a meteorite sample depend on: (1) the solvent type, (2) the extraction temperature, and (3) the duration of the extraction process. In order to optimize an extraction protocol for meteorite samples, each of the above factors should be tested on a suite of meteorite powders and procedural blanks, and monitored for total yields, reproducibility, potential reaction by-products when derivatization steps are used, and isotopic fractionation (if stable isotopes are measured). In practice, meteorite powders are not commonly accessible for method development. Industrial sediments [131] and other organic-rich geologic samples [49,132] have been used in some studies as alternative mineral matrices; however, the lack of a suitable meteorite analog on Earth for these purposes makes method development and optimization more challenging. The influence of individual factors (solvent, temperature, and time) have not been fully assessed for most soluble organic compound classes. For amino acid studies, the extraction conditions have remained consistent over time (aqueous extraction at ~100–110 °C for 18–24 h; Table 1). Likewise, extractions of meteoritic amines are generally carried out in water at 100–110 °C for 24 h (Table 2). However, extraction methods for other polar soluble organics such as monocarboxylic acids, aldehydes, and ketones, have been more variable and demonstrate the potential impact of differing protocols (Table 3 and Table 4). For instance, different yields of aliphatic aldehydes and ketones recently obtained by separate analyses of the same Murchison meteorite specimen may be partially explained by differing extraction procedures (DCM (100 °C for 24 h) versus aqueous extraction (100 °C for 24 h) [66,121]).

Successive solvent extractions of meteorite powders have been shown to yield larger abundances of soluble organics [114,115,133]. Simply increasing the temperature and duration of the extraction step has been shown to produce a similar effect (Figure 3). This continuous release of soluble organic matter during successive, longer, and warmer extractions indicates that the compounds may be bound to mineral substrates (e.g., chemisorption with phyllosilicate minerals) or as larger organic polymers, or synthesized in solution during the solvent extraction step. Post-extraction sample processing can also influence the yield of soluble organics. For instance, acid-vapor hydrolysis for amino acid analyses can have a major influence on abundances, in many cases doubling the yield (e.g., [50,83,84]). Increasing the temperature and duration of acid-hydrolysis results in higher amino acid recoveries (Figure 3), but can also lead to significant amino acid racemization. Recognizing this distinction between meteoritic soluble organics that are readily solvent-extractable versus organic byproducts of prolonged solvent extractions/sample processing steps is important to consider for our assessment of the organic inventory of asteroids in our solar system. The more readily extractable organics may be more representative of the types of organics involved in low-temperature aqueous alteration-driven reactions within parent body asteroids. Keeping the duration and temperature of extractions and hydrolyses consistent across studies is also important for maintaining consistency among study comparisons.

#### 5.2.2. Influence of Derivatization and Chromatographic Techniques

Several different derivatization and chromatographic techniques have been tested for meteoritic amino acid analyses (Table 1), though potential influences of all techniques have not been fully assessed. A method comparison study of a single Murchison specimen [124] demonstrated that the two most commonly applied amino acid methods (TFA derivatization for GC analysis vs. OPA/NAC derivatization for LC analysis) produced some slight differences in yields, with the GC analysis yielding ~50% higher abundance of α-AIB, and the LC analysis yielding ~3 times higher abundance of β-alanine. This type of method comparison is critical for validating cross-comparisons between studies. Some similarly variable results have been observed for other classes of soluble organic compounds in meteorites using differing chromatographic techniques (e.g., GC vs. LC analysis of nucleobases [69,71]). For the relatively volatile soluble organics discussed in this review (amines, monocarboxylic acids, aldehydes, and ketones), very few methodologies have been tested and the effects of each technique are poorly understood. Recent findings have shown that small changes to the derivatization protocol may have a significant impact on the types and abundances of meteoritic organics detected. For example, analyses of monocarboxylic acids as (*S*)-1-phenylethyl esters have yielded a substantially higher total abundance of monocarboxylic acids compared to other techniques [65], and analyses of monocarboxylic acids as (*S*)-2-methylbutyl ester derivatives have yielded a particularly high relative abundance of acetic acid (91% of the total monocarboxylic acid content) compared to other studies (13–28% of the total) [79] (Table 3). Similarly, differing techniques used for aldehyde and ketone analyses have been shown to produce notably distinct results. DCM extraction and DMB derivatization yielded a relatively higher abundance of formaldehyde, relatively ^13^C-enriched isotopic values for acetaldehyde and propionaldehyde and relatively ^13^C-depleted isotopic compositions for acetone and butyraldehyde, compared to measurements obtained via aqueous extraction and derivatization with PFBHA [66,114,121]. Thus, it may be advisable that a common methodology be used for analysis in order to allow direct comparisons of organics across meteorites, and direct comparisons of the same meteorite obtained through different sources. 

## 6. Conclusions

This review compiles and summarizes the range of analytical techniques applied to studies of extraterrestrial amino acids and structurally related amines, monocarboxylic acids, aldehydes, and ketones, to help facilitate data comparisons between studies, and to provide guidance for method selections for future sample analyses. Potential sources of variability among datasets are discussed, including compositional heterogeneity of meteorite samples, and the influences of differing extraction and purification protocols, derivatization methods, and chromatographic separation techniques. With these factors in mind, some potential considerations to implement for future analyses may include: (1) maintaining consistency in methodologies applied across studies to allow for more informative cross-comparisons of datasets; (2) carrying out direct comparisons of methodologies during method development experiments in order to gain a better understanding of the various effects of different analytical techniques; and (3) reporting detailed meteorite sample information for each soluble organic study to track compositional variability within single meteorite specimens.

## Figures and Tables

**Table 1:** Analyses of meteoritic amino acids.

Analyzed as	Extraction and Analytical Methodology	Author (Year Published)	Meteorites Analyzed
TFA alkyl (methyl, *n*-butyl, *sec*-butyl, (*S*)-*sec*-butyl, *sec*-pentyl, or isopropyl) ester derivatives	Meteorite powder heated or refluxed in water for 8–24 h at 110–110 °C. Aqueous portion removed, dried, optionally acid-hydrolyzed, desalted using cation exchange resin, and derivatized for GC, GC-MS and/or GC-IRMS analysis.	Kvenvolden et al. (1970, 1971) [13,134], Oró et al. (1971) [135,136], Lawless et al. (1971, 1972) [137,138], Lawless and Peterson (1975) [139], Pereira et al. (1975) [108], Kotra et al. (1979) [140], Shimoyama et al. (1979, 1985) [141,142], Cronin et al. (1981, 1985) [16,143], Peltzer et al. (1984) [45], Shimoyama and Harada (1984) [144], Cronin and Pizzarello (1986, 1997) [106,145], ^a^ Engel et al. (1990) [97]; ^a^ Engel and Macko (1997) [98]; Pizzarello and Cronin (2000) [92], Pizzarello and Cooper (2001) [146], Shimoyama and Ogasawara (2002) [147], Pizzarello et al. (2003, 2004, 2008, 2012) [18,74,76,148], Pizzarello and Huang (2005) [149], Pizzarello and Holmes (2009) [114], Monroe and Pizzarello (2011) [115], Chan et al. (2012) [150], Martins et al. (2015) [72], Koga and Naraoka (2017) [151], Pizzarello and Yarnes (2018) [117]	**CI1**: Orgueil [136,138]; **CM2**: ALH 77306 [140], Mukundpura [117], Murchison [13,16,18,45,92,97,98,106,108,134,135,139,143,145,146,147,148,149,151], Murray [92,136,137,146,148,149], Paris [72], Yamato 74662 [141], 791198 [142,147], 793321 [144]; **CR1**: GRO 95577 [74]; **CR2**: GRA 95229 [76,114], LAP 02342 [114], MIL 07525 [74], GRO 03116 [74], EET 92042 [74], GRA 06100 [74], PCA 91082 [74]; **CR3**: QUE 99177 [74], MET 00426 [74]; **C2_ung_**: Bells [115], Belgica 7904 [144]; **CO3**: Colony [150], Ornans [150]; **CV3**: Mokoia [136]; **LL3**: Chainpur [150], Bishunpur [150]
OPA/NAC, OPA/IBLC and OPA/IBDC-derivatives	Meteorite powder extracted in water for 6–24 h at 100–110 °C. Aqueous portion removed, dried, optionally acid-hydrolyzed, desalted using cation exchange resin, and derivatized for HPLC-FD and/or TOF-MS analysis.	Zenobi et al. (1992) [152], Brückner et al. (1994) [153], McDonald and Bada (1995) [53], Bada et al. (1998) [48], Glavin et al. (1999, 2006, 2010, 2011, 2012) [49,50,83,122,154], ^b^ Hutt et al. (1999) [155], Ehrenfreund et al. (2001) [17], Glavin and Bada (2001) [156], Botta et al. (2002, 2007, 2008) [157,158,159], Kminek et al. (2002) [160], Martins et al. (2007) [161,162], Glavin and Dworkin (2009) [163], Elsila et al. (2011) [57], Herd et al. (2011) [77], Burton et al. (2011, 2012, 2013, 2014, 2015) [51,52,84,164,165,166], Callahan et al. (2013, 2014) [126,167], Aponte et al. (2014) [82], Chan et al. (2018, 2019) [168,169], Friedrich et al. (2019) [125]; Simkus et al. (2019) [119]	**CI**: Ivuna [17,157,165], Orgueil [17,122,157,158,161,163], Y-86029 [165], Y-980115 [165]; **CM1**: ALH 88045 [158], MET 01070 [122,158], LAP 02277 [158], SCO 06043 [122]; **CM1/2**: ALH 83100 [50]; **CM2**: Murchison [17,50,52,57,82,122,125,126,153,155,156,157,158,161,163], Murray [17], Nogoya [157], Mighei [157], LEW 90500 [50,122,163], LON 94102 [122,163], Sutter’s Mill [52]; **CR**: Shişr 033 [161]; **CR1**: GRO 95577 [122,162]; **CR2**: Renazzo [157], EET 92042 [122,162,163], GRA 95229 [162]; **CR3**: QUE 99177 [122,163]; **C2_ung_**: Essebi [157], Tagish Lake [157,160,169], Tagish Lake 5b, 11h, 11i [77,83]; Tagish Lake 1, 4, 10a [119]; **CO3**: ALH 77307 [164], MIL 05013 [164], DOM 08006 [164]; **CH3**: ALH 85085 [84], PCA 91467 [84], PAT 91546 [84]; **CBb**: MAC 02675 [84]; **CB**: MIL 05082 [84], MIL 07411 [84]; **CV3**: Allende [157], ALH 84028 [164], EET 96026 [164], LAP 02206 [164], LAR 06317 [164], GRA 06101 [164]; **CK3**: NWA 5956 [166]; **CK4**: LAR 04318 [166], ALH 85002 [166]; **CK4/5**: PCA 82500 [166]; **CK5**: LAP 03784 [159], EET 92002 [166]; **CK6**: LEW 87009 [166], LAR 06872 [166]; **R3**: LAP 03834 [166]; **R4**: LAP 031135 [166]; **LL5**: LAP 03624 [159], LAP 03573 [159], LAP 03637 [159]; **L6**: Shişr 031 [161], Shişr 035 [161]; **H3-6**: Zag halite crystals [168]; **Ureilite**: Almahata Sitta [51,154], ALH 77257 [164], EET 83309 [164], LEW 85328 [164], GRA 95205 [164], LAR 04315 [164]; **H4**: LAP 03553 [159], Forest Vale [152]; **Lunar**: MAC 88105 [48]; **Martian**: EET 79001 [53], ALH 84001 [48], Nakhla [49], RBT 04262 [167]
OPA/NAC derivatives	Meteorite powder extracted overnight at room temperature in 1 M or 6 M HCl. Supernatant separated, dried, optionally acid-hydrolyzed, desalted using cation exchanged resin, then derivatized for HPLC analysis.	Bada et al. (1998) [48], Glavin et al. (1999) [49]	**Lunar**: MAC 88105 [48]; **Martian**: ALH 84001 [48], Nakhla [49]
Ninhydrin derivatives	Meteorite powder refluxed in water for 20–24 h. Aqueous portion separated, dried, re-dissolved in water and split in half for acid-hydrolyzed and unhydrolyzed analyses using LC.	Cronin and Moore (1971,1976) [85,170], Cronin (1976) [81,171]	**CM2**: Murchison [81,85,171], Murray [85], Nogoya [170]; **CV3**: Allende [85], Mokoia [170]
Bulk underivatized amino acids	Meteorite powder refluxed in water at 100–110 °C for 24 h. Aqueous portion removed, dried, acid hydrolyzed, and then desalted using cation exchange resin for IRMS.	Epstein et al. (1987) [172], Pizzarello et al. (1991, 1994) [113,173]	**CM2**: Murchison
PFP alkyl ((*S*)-*sec*-butyl) ester derivatives	Meteorite powder refluxed in water at 100 °C for 8 h. Aqueous portion separated by filtration, dried in a rotary evaporator, hydrolyzed, dried and desalted using cation exchange resin for GC-MS or GC-IRMS analyses.	Engel and Nagy (1982) [96]	**CM2**: Murchison
PFP ester derivatives	Meteorite powder refluxed in water at 100 °C for 6 h. Aqueous portion separated, dried, acid hydrolyzed, and derivatized for GC-MS analysis.	Hilts et al. (2014) [70]	**C2_ung_**: Tagish Lake 5b, 11h, 11i
MTBSTFA derivatives	Meteorite powder stirred in water for 46 h with intermittent sonication. Aqueous portion concentrated by rotary evaporation, treated with ethanol for removal of salts, then acidified and desalted using cation exchange resin for GC-MS analysis.	Cooper and Cronin (1995) [47], Lerner and Cooper (2005) [174]	**CM2**: Murchison
ECEE or ECHFBE derivatives	Meteorite powder extracted in water at 100 °C for 20 h. Aqueous portion was separated by centrifugation, split for acid-hydrolyzed and unhydrolyzed analyses, dried for derivatization and multidimensional (GC x GC) analysis.	Meierhenrich et al. (2004) [94], Myrgorodska et al. (2016) [95]	**CM2**: Murchison
PBSE derivatives	Meteorite powder extracted via subcritical water extraction at 200 °C and 3000 MPa with 5 min equilibration time. Portion of the extract was diluted, derivatized and analyzed using micellar electrokinetic chromatography.	Chiesl et al. (2009) [99]	**CM2**: Murchison
OPA derivatives	Meteorite powder in water at 110 °C or at room temperature for 24 h. Aqueous portion separated by filtration, dried in a rotary evaporator, acid hydrolyzed, dried, and re-dissolved in aqueous sodium citrate for liquid chromatography.	Cronin et al. (1979) [175], Matrajt et al. (2004) [24]	**CM2**: ALH 77306 [175]; Murchison [24]; **CO3**: ALH 77307 [175]; South Pole Water Well micrometeorites [24]

For studies where more than one derivatization method was used, the methodology used for quantification is listed, unless otherwise noted. ^a^ A portion of the aqueous extract was analyzed by HPLC for quantification. Only the methodology used for GC and GC-IRMS analysis is described here. ^b^ Amino acids were also analyzed using a microchip capillary electrophoresis technique. Only the methodology used for LC analysis is described here. TFA: trifluoroacetic anhydride; OPA: *o*-phthalaldehyde; NAC: *N*-L-acetyl cysteine; IBLC: *N*-isobutyryl-l-cysteine; IBDC: *N*-isobutyryl-d-cysteine; PFP: *N*-pentafluoropropionic anhydride; MTBSTFA: *N*-methyl-*N*-(*tert*-butyldimethylsilyl)-trifluoroacetamide with 1% *tert*-butyldimethylsilyl chloride; ECEE: *N*-ethoxycarbonyl ethyl esters; ECHFBE: *N*-ethoxycarbonyl heptafluorobutyl ester; PBSE: Pacific Blue succinimidyl ester.

**Table 2:** Compound-specific analyses of meteoritic amines.

Analyzed as	Extraction and Analytical Methodology	Author (Year Published)	Meteorites Analyzed
Underivatized amines, DNP derivatives, and OPA derivatives	Meteorite powders refluxed for 24 h. Aqueous portion removed after centrifugation, then dried and acid-hydrolyzed. Separate set of meteorite powders extracted in water at 120 °C for four days with no subsequent hydrolysis step. Analyzed by GC-MS and ion exchange chromatography	Jungclaus et al. (1976) [15]	**CM2**: Murchison
PFP and OPA derivatives	Meteorite powders extracted in triple-distilled water at 110 °C for 24 h. Aqueous portion removed after acidification and cryogenic transfer. Residue taken to pH > 12.5 using NaOH, frozen and acidified for cryogenic transfer of volatile amines for GC-MS and ion exchange chromatography	Pizzarello et al. (1994) [113]	**CM2**: Murchison
TFA derivatives (amides)	Meteorite powder put in degassed glass vial with triple-distilled water at 100 °C and intermittent sonication for 20 h. Aqueous extract was made acidic and concentrated, then it was made basic and cryogenically transferred, acidified, dried in rotary evaporator, and derivatized for GC-MS analysis	Pizzarello et al. (2008) [76]	**CR2**: GRA 95229
TFA derivatives (amides)	Meteorite powder put in degassed glass vial with triple-distilled water at 100 °C and intermittent sonication for 20 h. Aqueous portion separated by centrifugation, dried completely, acid hydrolyzed, re-dried and re-dissolved in water, made basic, cryogenically transferred, acidified, dried in rotary evaporator, and derivatized for GC-MS analysis	Pizzarello and Holmes (2009) [114]	**CR2**: LAP 02342
TFA derivatives (amides)	Meteorite powders extracted in water at 25 °C for 24 h, followed by a 100 °C 24 h extraction. Aqueous portions separated after each extraction period were made basic, cryogenically transferred, acidified, dried in rotary evaporator, and derivatized for GC-MS analysis	Monroe and Pizzarello (2011) [115]	**C2_ung_**: Bells; **CI1**: Ivuna
TFA derivatives (amides)	Meteorite powder put in degassed glass vial with triple-distilled water at 100 °C for 20 h. Aqueous portion separated by decantation, concentrated, basified, cryogenically transferred, acidified, dried in rotary evaporator, and derivatized for GC-MS analysis	Pizzarello et al. (2012) [74], Pizzarello and Yarnes (2016, 2018) [116,117]	**CM2**: Mukundpura [117]; **CR1**: GRO 95577 [74]; **CR2**: GRA 95229 [116]; LAP 02342 [116]; MIL 07525 [74,116], PCA 91082 [74,116], EET 92042 [74,116]; **CR3**: QUE 99177 [74,116], MET 00426 [74,116]
TPC derivatives (amides)	Meteorite powders extracted in water at 100 °C for 24 h. Aqueous extracts separated after centrifugation were acidified and taken to dryness. Dry residues were re-dissolved in water and treated with NaOH, then centrifuged and the aqueous fractions separated from precipitate were re-acidified and dried completely. The residues were dissolved in diluted aqueous NaOH, extracted using DCM and derivatized for GC-MS and GC-IRMS analyses.	Aponte et al. (2014, 2015, 2016, 2017) [82,176,177,178,179]	**CI1**: Orgueil [176]; **CM1/2**: ALH 83100 [177]; **CM2**: Murchison [82], LEW 90500 [177], LON 94101 [177]; **CR2**: MIL 090001 [179], LAP 02342 [177], GRA 95229 [177]; **CO3**: DOM 08006 [178], MIL 05013 [178]; **CV3**: Allende [178], LAP 02206 [178], GRA 06101 [178]; **CK4**: ALH 85002 [178]; **CK5**: EET 92002 [178]

DNP: 2,4-dinitrophenyl; OPA: *o*-phthalaldehyde; PFP: *N*-pentafluoropropionic anhydride; TFA: trifluoroacetic anhydride; TPC: (*S*)-(–)-N-(trifluoroacetyl)pyrrolidine-2-carbonyl chlorid.

**Table 3:** Compound-specific analyses of meteoritic monocarboxylic acids.

Analyzed as	Extraction and Analytical Methodology	Author (Year Published)	Meteorites Analyzed
Methyl esters	Meteorite powder refluxed in glass flask or in degassed glass tube using 1%, 5% or 10% KOH/MeOH for 3 h. Water added to extract for DCM or benzene partition. Aqueous fraction taken to dryness, re-dissolved in water, acidified, and DCM-extracted for GC-MS analysis	Yuen and Kvenvolden (1973) [61], Shimoyama et al. (1986, 1989) [63,180], Naraoka et al. (1999) [64]	**CM2**: Murchison [61], Murray [61], and Asuka 881280 [64], 881334 [64], 881458 [64]; **C2**: Yamato 791198 [63], 74662 [180] and 793321 [180]
Methyl esters	Meteorite powder refluxed in 6% KOH/MeOH for 3 h. Aqueous portion separated by decantation, solution taken to dryness, acidification, filtration, and diethyl ether-extraction for GC and GC-IRMS analyses	Lawless and Yuen (1979) [62]	**CM2**: Murchison
Underivatized MCAs	Meteorite chips put in degassed flask with deionized water, disaggregation of chips by freeze-thaw cycles and sonication. Aqueous portion separated, neutralized, taken to dryness by rotary evaporation, re-dissolved in water, and vacuum distilled for suppression chromatography and GC-IRMS analyses	Yuen et al. (1984) [118]	**CM2**: Murchison
Underivatized MCAs	No details provided about extraction protocol. Analysis of MCAs performed using ion exclusion chromatography	Briscoe and Moore (1993) [181]	**CM2**: Murchison; **CV3**: Allende; **LL3**: Parnallee; **L6**: Leedey; **E4**: Abee
Underivatized MCAs	Meteorite chips or powder extracted in degassed glass tube or round-bottom flask with ultrapure deionized or double-distilled water at 100 or 110 °C for 6 or 24 h. Aqueous extract taken to pH > 10, concentrated by rotary evaporation, acidified for SPME, GC-FID, GC-MS and GC-IRMS analyses	Huang et al. (2005) [75], Aponte et al. (2011) [78], Herd et al. (2011) [77], Dillon et al. (2013) [80], Hilts et al. (2014) [70]; Simkus et al. (2019) [119]	**CM2**: Murchison [75,78], ALH 84033 [78], Sutter’s Mill [80], WIS 91600 [78]; **C2_ung_**: Tagish Lake 5b, 11h, 11i and 11v [70,77]; Tagish Lake 1, 4, 10a [119]; **CM1**: ALH 84034 [78]; **CR2**: EET 87770 [78]; **CV3**: MET 00430 [78]
Underivatized MCAs	Meteorite powder put in degassed glass vial with triple-distilled water at 100 °C and intermittent sonication for 20 h. Aqueous extract taken to pH = 11.5, concentrated by rotary evaporation, acidified, cryogenically transferred and DCM-extracted for GC-MS analysis	Pizzarello et al. (2008) [76]	**CR2**: GRA 95229
Underivatized MCAs	Meteorite powder put in degassed glass vial with distilled water at 100 °C for 20 h or at 25 °C for 24 h, followed by a 100 °C 24 h extraction. Aqueous portions separated after each extraction period were made acid, cryogenically transferred, acidified, and DCM-extracted for GC-MS analysis	Monroe and Pizzarello (2011) [115]	**CI1**: Ivuna; **C2_ung_**: Bells
Underivatized MCAs	Meteorite powder put in degassed glass vial with distilled water at 100 °C for 20 h. Aqueous portions separated after each extraction period were made acid, cryogenically transferred, acidified, and DCM-extracted for GC-MS analysis	Pizzarello et al. (2012) [74], Pizzarello and Yarnes (2018) [117]	**CM2**: Mukundpura [117]; **CR1**: GRO 95577 [74]; **CR2**: MIL 07525 [74], PCA 91082 [74]; **CR3**: QUE 99177 [74], MET 00426 [74]
Underivatized MCAs and as (*S*)-1-phenylethyl esters	Meteorite powder put in Teflon tube containing aqueous 1 N NaOH, 30 min sonication, stirring for 2 h at room temperature. Acidification of aqueous extract and DCM-partition for GC-FID and GCMS underivatized analyses, and esterification for GC-MRM and GC-IRMS analyses	Aponte et al. (2014) [65]	**CM2**: Murchison, LON 94101
Derivatized as (*S*)-2-methylbutyl esters	Meteorite powder extracted in distilled water at 100 °C for 24 h. Aqueous portion separated, MgCl_2_ solution added, and solution taken to dryness by centrifugal evaporation. Acid-catalyzed esterification of residue for GC-MS and GC-IRMS analyses	Aponte et al. (2019) [79]	**CM1/2**: ALH 83100; **CM2**: Murchison, LEW 90500, LON 94101, EET 96029; **CR2**: MIL 090001, LAP 02342, GRA 95229, MIL 090657; **CO3**: DOM 08006, MIL 05013; **CV3**: Allende, LAP 02206, GRA 06101; **CK4**: ALH 85002, **CK5**: EET 92002

**Table 4:** Compound-specific analyses of meteoritic aldehydes and ketones.

Analyzed as	Extraction and Analytical Methodology	Author (Year Published)	Meteorites Analyzed
Underivatized carbonyls for GC-MS MBTH derivatives (azines) for colorimetric	Meteorite powder extracted in water at 120 °C for 4 days. Aqueous portion removed after centrifugation for head space GC-MS of volatile species, followed by derivatization for colorimetric analyses, and removal of derivatization tag for GC-MS re-analysis	Jungclaus et al. (1976) [14]	**CM2**: Murchison, Murray
PFBHA derivatives (oximes)	Meteorite powders extracted in water at 80 °C for 2 h, then at 80 °C for 20 h, then at 100 °C 24 h. Aqueous portions separated after each extraction period were derivatized (35 °C, 2 h), then acidified. PFBHA derivatives were extracted from solution in hexane for GC-MS analysis	Pizzarello and Holmes (2009) [114]	**CM2**: Murchison; **CR2**: GRA 95229, LAP 02342
PFBHA derivatives (oximes)	Meteorite powders extracted in water at 25 °C for 24 h, followed by a 100 °C 24 h extraction. Aqueous portions separated after each extraction period were derivatized (35 °C, 2 h), acidified, and extracted using hexane for GC-MS and GC-IRMS analyses	Monroe and Pizzarello (2011) [115]	**CI1**: Ivuna; **C2_ung_**: Bells
PFBHA derivatives (oximes)	Meteorite powders extracted in water at 100 °C for 24 h. Aqueous portions were derivatized (35 °C, 2 h), acidified, and extracted using hexane for GC-MS analysis	Pizzarello et al. (2012) [74]	**CR1**: GRO 95577; **CR2**: MIL 07525, PCA 91082; **CR3**: QUE 99177, MET 00426
PFBHA derivatives (oximes)	Meteorite powders extracted in water at 100 °C for 24 h. Aqueous portions were derivatized (room temperature, 24 h), acidified, and extracted using DCM for GC-MS and GC-IRMS analyses	Simkus et al. (2019) [119,121]	**CM2**: Murchison [121]; **C2_ung_**: Tagish Lake 1, 4, and 10a [119]
DMB derivatives (acetals)	Meteorite powders extracted in DCM at 100 °C for 24 h. DCM portions were derivatized for GC-MS and GC-IRMS analyses	Aponte et al. (2019) [66]	**CI1**: Orgueil; **CM1/2**: ALH 83100; **CM2**: Murchison, LEW 90500, LON 94101, EET 96029; **CR2**: MIL 090001, LAP 02342, GRA 95229, MIL 090657; **CV3**: Allende

MBTH: 3-methyl-2-benzothiazolone hydrazone hydrochloride; PFBHA: 2,3,4,5,6-pentafluorobenzyl hydroxylamine; DMB: (*S*,*S*)-(–)-1,4-dimethoxy-2,3-butanediol.
